# Shifting the Spotlight From the Mandibular Condyle to the Coronoid Process: A Report of a Unique Case of Trifid Mandibular Coronoid Process

**DOI:** 10.7759/cureus.37593

**Published:** 2023-04-14

**Authors:** Archna Nagpal, Anusha Vaddi, Aditya Tadinada

**Affiliations:** 1 Oral and Maxillofacial Radiology, University of Connecticut, Farmington, USA; 2 Oral and Maxillofacial Radiology, Virginia Commonwealth University School of Dentistry, Richmond, USA

**Keywords:** cone beam computed tomography, incidental, volume rendering, mandible, coronoid, trifid

## Abstract

The trifid mandibular coronoid process is an uncommon finding characterized by three projections arising from the mandibular ramus instead of a single triangular coronoid process. Previous authors reported cases of the bifid coronoid process. The authors referred to them as the bifid/second/double coronoid process. This article aims to report a unique case of a trifid coronoid process incidentally detected during radiographic evaluation for implant planning. This article also emphasizes the effectiveness of cone beam computed tomography (CBCT) volume rendering as a valuable tool in demonstrating morphological variations such as the trifid coronoid process. In addition, we discussed possible etiologies for the trifid coronoid process. To the best of our knowledge, this is the first case of the trifid coronoid process.

## Introduction

The mandible is the largest mobile bone of the facial skeleton that lodges lower dentition [1]. The mandible is a curved bone that has two major parts: the mandibular body and the rami on either side of the body. The ramus is the vertical part that unites to the body near the angle of the mandible. The superior aspect of the ramus has two processes named coronoid and condylar process [2]. The coronoid process of the mandible is a triangular plate of bone that projects upward and slightly forward [1]. It develops endochondrally from the primary ossification center, separates from Meckel’s cartilage during the initial developing stages, and later gets integrated into the ramus of the mandible in the eighth to ninth week of gestation [3]. The coronoid process provides attachment to two important muscles of mastication: the temporalis and masseter [2]. These muscles play a key role in functional movements such as mastication, swallowing, and speech, thus emphasizing the role of the coronoid process in morpho-functional dependence. The condylar process forms a part of the temporomandibular joint (TMJ). It gives attachment to the lateral pterygoid muscle. Most of the literature has been focused on reporting anomalies of the condylar process such as “bifid condyle,” “trifid condyle,” and “tetrafid condyle” [4]. The reporting of these variations of the mandibular condyle has increased with the advent of advanced imaging techniques, especially computed tomography (CT), cone beam computed tomography (CBCT), and magnetic resonance imaging (MRI) [4,5].

The coronoid process demonstrates variations in its morphology such as shape, width, and length. They can be either triangular, rounded, or hook-shaped [6]. The abnormal elongation of the mandibular coronoid process, formed of histologically normal bone, is called coronoid hyperplasia. This might lead to difficulty in mastication and a decreased range of motion of the TMJ [2,7]. Goh et al. performed a review including all the case reports and case series on coronoid hyperplasia and suggested a crucial role of the temporalis muscle in the pathogenesis of coronoid hyperplasia either directly or indirectly [6]. This condition leads to the impingement of the coronoid process on the zygomatic bone restricting mandibular movements [6]. Coronoid hypoplasia is a defective or underdeveloped coronoid process. It is a rare entity that is usually considered as a developmental abnormality and can also be associated with Melnick-Needles syndrome, which is an osteodysplasia, characterized by generalized bony alterations. It has also been suggested that coronoid hypoplasia is due to the activity of the temporalis muscle in prenatal and early postnatal life, but it can occur with unchanged muscle activity also [8].

Another anomaly of the coronoid process is the formation of a new joint between a pathologically elongated coronoid process and the body of the malar homolateral bone, known as Jacob’s disease [9]. The possible causes for this condition are temporalis muscle hyperactivity, trauma, endocrine stimuli, and genetic alteration. It is associated with coronoid hyperplasia, in which the coronoid process forms a joint with the inner surface of the malar bone and is accompanied by cartilaginous structures and the formation of a synovial capsule. Raccampo et al. did an extensive literature review on 116 cases, including the three cases of Jacob’s disease that they added. They found that patients usually remain relatively asymptomatic in the initial stages, followed by the limitation of mandibular movements and worsening reduction in maximal mouth opening. This insidious clinical onset may be mistaken for temporomandibular disorders leading to incorrect treatment [10]. Güven reported a case of zygomatico-coronoid ankylosis in a 14-year-old female with limited mouth opening following trauma. CT scan revealed a joint-like structure between the zygomatic arch and coronoid process [11].

The presence of two or three individual coronoid processes in place of a single mandibular coronoid process is an unusual feature. The terms bifid and trifid were derived from the Latin word meaning a cleft into two and three parts [4]. The bifid or trifid coronoid process implies the presence of two or three coronoid processes separated by cleft or grooves of variable depths. There are limited cases in the literature reporting variation in the number of the coronoid process such as the bifid coronoid process. Patil et al. [2], Cranin [12], Lundquist and Wege [13], Kansu et al. [14], and de Gittins et al. [15] reported cases of bifid/double coronoid process or second coronoid process. To date, no case of trifid coronoid process has been reported.

Other pathologies seen in association with the coronoid process are coronoid exostosis, coronoid osteochondroma, osteomas, pseudo-ankylosis, trauma, foreign body reaction, or irradiation [16].

Most of the conditions and pathologies associated with the coronoid process have been underestimated and underreported. Anatomically, the coronoid process lies in the vicinity of the zygomatic bone, and the coronoid defects might cause the limitation of mouth opening due to their impingement against the medial surface of the zygoma during mandibular movements. The diagnosis of these disorders may be limited since in patients with a reduced mouth opening, the attention is usually directed toward the TMJ and mandibular condyle [16]. Conventional two-dimensional radiographs of the mandible do not depict the coronoid process due to the superimposition of overlying structures. With the advent of three-dimensional (3D) imaging techniques, especially CBCT, the diagnosis of coronoid process abnormalities has become more achievable.

We report a unique case of the trifid coronoid process that was incidentally detected during the radiographic evaluation for implant planning. This article focuses on describing imaging features of trifid coronoid process and discussing possible etiology.

## Case presentation

**Figure 1:**
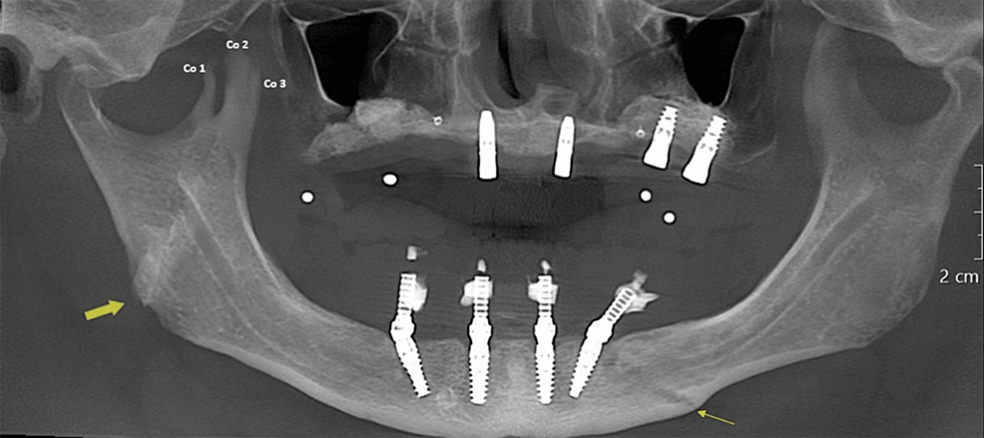
CBCT panoramic reconstruction demonstrating right the trifid coronoid process and oblique fracture lines in the right angle/ramus (bold yellow arrow) and the left parasymphysis (yellow arrow) region CBCT, cone beam computed tomography; Co1, coronoid 1; Co2, coronoid 2; Co3, coronoid 3

A 60-year-old male patient reported to the University of Connecticut School of Dental Medicine for dental implant therapy. A cone beam computed tomography (CBCT) scan was acquired for treatment planning. There was no obvious facial deformity or asymmetry. Upon image reformatting, abnormal morphology of the right coronoid process was identified. The panoramic reconstruction showed three projections arising from the right coronoid process and an oblique area of increased radio-opacity at the right mandibular angle region with a slanting radiolucent line at the left parasymphysis region (Figure 1).

**Figure 2:**
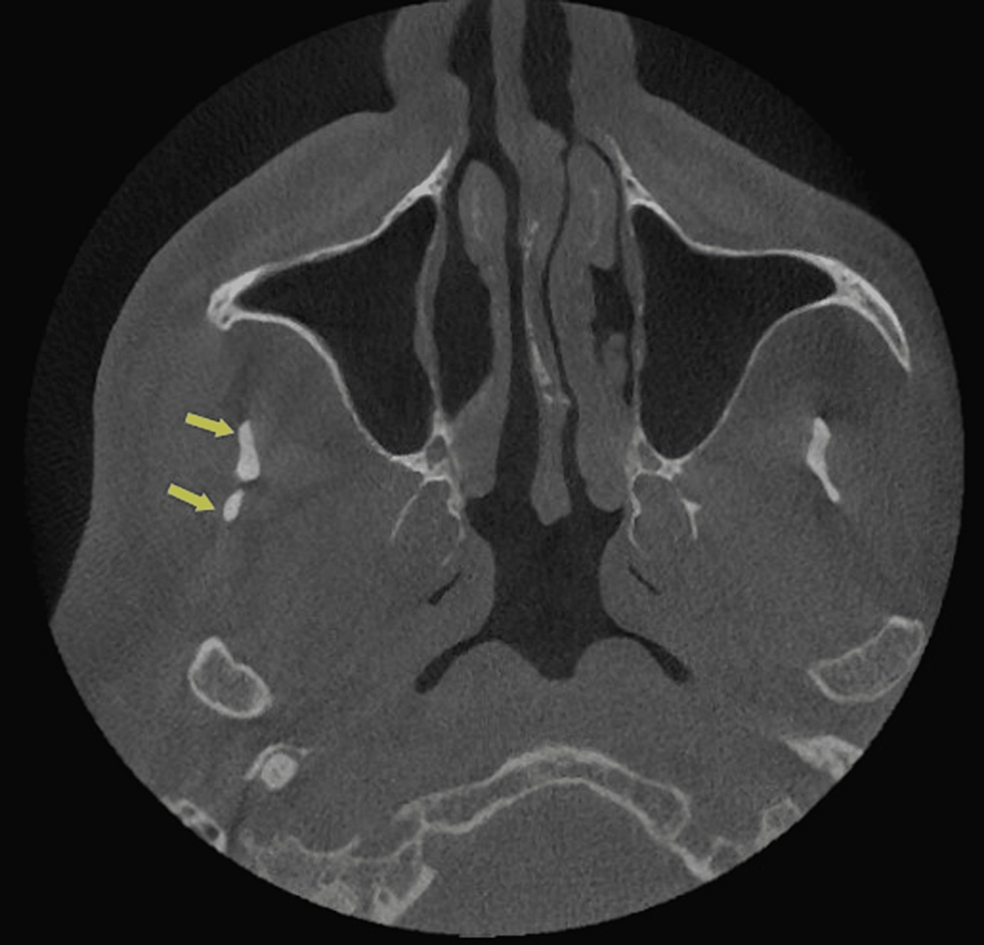
CBCT axial projection showing two projections in the right coronoid process CBCT: cone beam computed tomography

In the axial section, the right coronoid process appeared as two projections (Figure 2).

There was no effect of the right coronoid process on the zygomatic arch radiographically. The posterior surface of the zygoma on the right side appeared normal. It was practically impossible to view all three triangular projections from the coronoid process in a single section even after carrying out exhaustive reformatting of the volume in the non-orthogonal planes.

**Figure 3:**
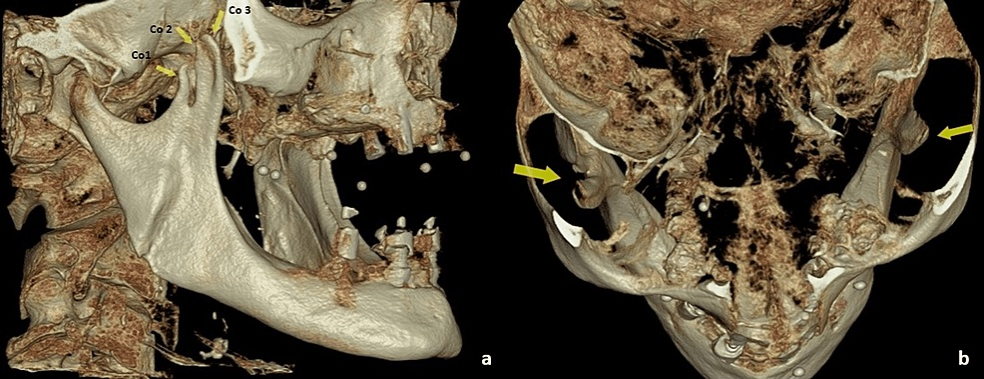
(a) 3D volume rendering (right lateral view) showing the right trifid coronoid process. (b) 3D volume rendering (superior view) showing the right trifid coronoid process and normal left coronoid process 3D, three dimensional; Co1, coronoid 1; Co2, coronoid 2; Co3, coronoid 3

The 3D volume rendering showed three triangular projections labeled as coronoid 1 (Co1), coronoid 2 (Co2), and coronoid 3 (Co3) in the region of the coronoid process giving an impression of a trifid coronoid process (Figure 3).

**Figure 4:**
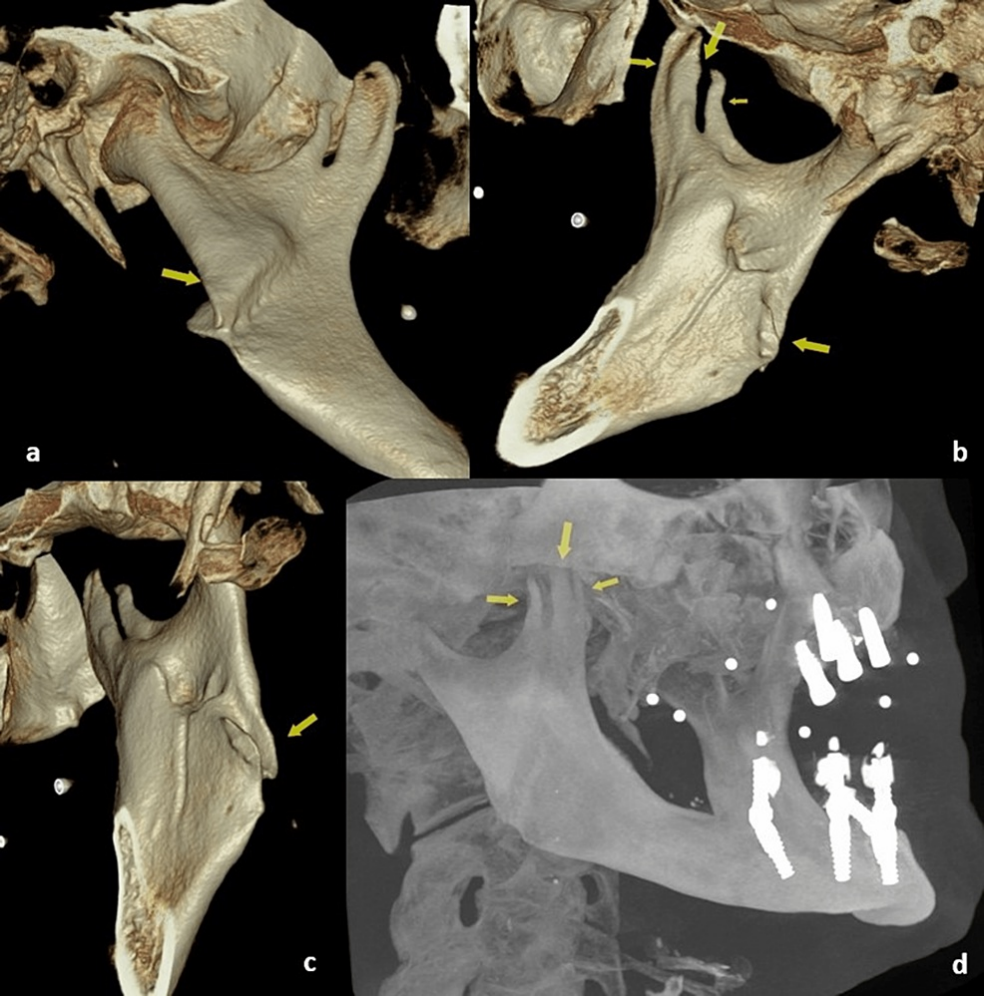
3D volume rendering with the region of interest edited showing the trifid coronoid process from different angles (a, lateral; b, medial; and c, posterior). (d) Maximum intensity projection (MIP) reconstruction of CBCT showing three heads of the right mandibular coronoid process 3D, three dimensional; CBCT, cone beam computed tomography

The Co1 was adjacent to the sigmoid notch and appeared to be completely separated from Co2 and Co3. The Co2 and Co3 were close to each other with a cleft separating them suggesting that they were individual projections. Many CBCT software have a feature to perform the segmentation of the user-defined region of interest in the volume-rendered images. The segmented image showed an oblique depression on the lateral surface of the angle of the mandible extending over the right ramus and ending between Co1 and Co2. The medial surface of the ramus revealed an oblique projection extending from the angle of the mandible to the cleft between Co1 and Co2. There was a step deformity at the angle of the right mandible (Figure 4).

These radiographic features suggested a previous oblique right angle/ramus fracture possibly traversing through the right coronoid process in an oblique pattern, with an overlap of fracture fragments followed by malunion. A thorough radiographic evaluation also revealed that none of the coronoid processes, Co1, Co2, and Co3, were close to the posterior surface of the zygoma. Since there was no difficulty in opening or closing the mouth or any restriction during mandibular jaw movements, we decided to periodically follow up the patient.

## Discussion

In the current case, three separate triangular projections were seen instead of a single coronoid process on the right side of the mandible. Each of these projections resembled an individual coronoid process, on CBCT panoramic reconstruction and volume-rendered images. These projections were separated by grooves of variable depths, hence justifying the term “trifid coronoid process.”

The exact etiology of the “bifid” or “trifid” coronoid process is not well known or understood. The radiographic features of the current case suggest a previous fracture traversing obliquely through the coronoid process leading it to split into parts rather than the complete separation of the coronoid process from the mandibular ramus with a concomitant fracture of the right angle/ramus and the left parasymphysis region. There is a clear indication of malunion as there is an overlap of fractured fragments. This finding is in agreement with Kansu et al. The authors reported a case of the bifid coronoid process in a 66-year-old female with a history of trauma to the area 30 years ago [14].

However, there was no prior history of trauma in other cases of bifid coronoid reported in the literature. Cranin reported an incidental finding of osseous protrusion from the sigmoid notch, referred to as the second coronoid process, on the mandibular right side in a 28-year-old female [12]. Lundquist and Wege reported two normally formed coronoid processes, referred to as the double coronoid process, in a 41-year-old white male on the right side of the mandible [13]. Patil et al. found a unilateral bifid coronoid process on the right side of the jaw in a 58-year-old male, which was an incidental finding on the panoramic image that was later confirmed by CBCT [2]. de Gittins et al. reported a case of the double coronoid process in a 61-year-old female on the left side of the jaw. They assumed that an error in embryogenesis was a cause for the duplication of the coronoid process [15].

The coronoid process provides attachment to the temporalis muscle; we assume that a trifid appearance of the coronoid process could be a result of malunion following trauma, strong muscular forces, and bone remodeling. The persistence of the cartilaginous growth center of the coronoid process can be thought of as a good possibility to justify the continued growth of the process [17]. In the current case, we hypothesize that it was a favorable fracture with muscles approximating the fracture fragments, rather than separating them apart from each other and adapting themselves to normal physiological movements of the mandible. There are three coronoid processes separated via grooves of variable depths; these multiple coronoid heads could be a consequence of continuous healing and remodeling.

Trauma has been proposed as one of the possible etiologies for a bifid/trifid mandibular condyle; following a fracture to the condylar neck, the force of the lateral pterygoid muscle displaces the condyle in an anteromedial direction. The metaplasia of local fibroblasts in the condylar neck produces a new condylar head at the normal anatomical site, while the original displaced condylar head undergoes resorption. As a result, the traumatized joint will have two or more condyles [5]. This is in concordance with our explanation regarding the formation of three coronoid processes following an episode of trauma.

Coronoid fractures are comparatively rare, accounting for less than 3% of all facial fractures. Since the coronoid process is located in a protected position under the zygoma, fractures of the coronoid process are predictably associated with high-velocity impact to the lateral midface and mandible [18]. This might account for the rare occurrence of the bifid and trifid coronoid processes.

One of the major consequences of enlarged coronoid process is impingement against the medial surface of the body of the zygomatic bone and progressive decreased mouth opening and facial asymmetry. In the current case, the patient did not have any major limitation in functional mandibular movements. All the three coronoid processes, Co1, Co2, and Co3, were not close to the medial surface of the zygomatic bone and hence did not impinge on it during mandibular movements. This may be an explanation of why our patient had no limitation of mouth opening. Ilguy et al. reported three cases of coronoid process hyperplasia and demonstrated the relationship between elongated mandibular coronoid process and the limitation of mouth opening with cone beam computed tomography. The authors stated that instead of the length, the distance between the coronoid process and the inner surface of the zygomatic bone may be the actual reason for the limitation of mouth opening [7]. The muscles attached to the coronoid process are temporalis and masseter, which aid in mandibular movements. The coronoid part of the masseter is attached at the posterior edge and the root of the coronoid process, and the temporalis is attached along the inferior anterior edge of the coronoid process [3]. Since there was no detachment of the coronoid process from the mandibular ramus, possibly, the functioning of the muscles was remarkably unaffected. Our patient is on periodic follow-up to assess any signs of any progressive decrease in the range of motion of the mandible.

A thorough radiographic evaluation is essential to arrive at an appropriate diagnosis of a coronoid process abnormality and a diligent follow-up. Conventional temporomandibular joint radiographs of the mandible do not often provide the needed information due to superimpositions and distortions [17]. With the advent of advanced imaging technology such as CBCT where the area of interest is majorly the maxillofacial area, the imaging of the coronoid process has become more achievable as opposed to plain radiography. It was nearly impossible to visualize all three coronoid processes in a single section in our patient. In the current case, the volume-rendering tool served as a useful aid to visualize the morphology of the coronoid and to explore it in different planes. In addition, volume rendering was very helpful in assessing the mandibular ramus.

## Conclusions

Although both condyle and coronoid are the processes arising from the ramus of the mandible and give attachment to the muscles of mastication, only the mandibular condyle has been extensively investigated. Morphological alterations regarding mandibular coronoid may be encountered during the routine radiographic examination; we emphasize a methodical assessment of CBCT volumes and urge all clinicians to evaluate the coronoid process during the analysis of this region. To the best of our knowledge, this is the first case of the trifid coronoid process reported in the literature. This case report also highlights the usefulness of CBCT volume rendering as a valuable tool in displaying the variations such as the trifid coronoid process.
